# Artificial Intelligence and the Ethical Foundations of Cardiothoracic Surgery: Evidence, Accountability, and the Limits of Delegated Judgment

**DOI:** 10.3390/medsci14050586

**Published:** 2026-09-18

**Authors:** Vasileios Leivaditis, Francesk Mulita, Vasiliki Androutsopoulou, Sofoklis Mitsos, Periklis Tomos, Ioannis Panagiotopoulos, Konstantinos Nikolakopoulos, Elias Liolis, Theodora Skoura, Efstratios Koletsis

**Affiliations:** 1 Department of Cardiothoracic and Vascular Surgery, Westpfalz Klinikum, 67655 Kaiserslautern, Germany; 2 Department of General Surgery, General University Hospital of Alexandroupolis, Medical School, Democritus University of Thrace, 68100 Alexandroupolis, Greece; 3 Department of Cardiothoracic Surgery, University Hospital of Larissa, 41110 Larissa, Greece; 4 Department of Thoracic Surgery, Attikon General Hospital, National and Kapodistrian University of Athens, 12462 Athens, Greece; sophocmit@yahoo.gr (S.M.);; 5 Department of Cardiac Surgery, Ippokrateio General Hospital of Athens, 11527 Athens, Greece; mdgiapan@yahoo.gr; 6 Department of Vascular Surgery, General University Hospital of Patras, 26504 Patras, Greece; 7 Department of Oncology, General University Hospital of Patras, 26504 Patras, Greece; 8 Medical School, National and Kapodistrian University of Athens (NKUA), Aretaeion Hospital, 11528 Athens, Greece; 9 Department of Cardiothoracic Surgery, General University Hospital of Patras, 26504 Patras, Greece; ekoletsis@hotmail.com

**Keywords:** artificial intelligence, cardiothoracic surgery, cardiac surgery, thoracic surgery, surgical judgment, accountability, informed consent, algorithmic bias, human oversight

## Abstract

Artificial intelligence (AI) is moving rapidly from retrospective prediction and image analysis into treatment selection, operative planning, intraoperative guidance, and postoperative prognostication in cardiothoracic surgery. This transition raises an ethical problem that cannot be resolved by model accuracy alone: when an algorithm begins to shape a high-stakes clinical decision, the distribution of knowledge, authority, and responsibility also changes. This review synthesizes cardiothoracic and closely related medical evidence available through August 2026, with emphasis on quantitative performance, human–AI interaction, bias, patient autonomy, and liability. The available evidence is simultaneously encouraging and cautionary. Machine-learning approaches can improve predictive performance and AI-assisted thoracic planning can reduce errors and increase procedural consistency; however, these gains have not consistently translated into superior patient outcomes. Human–AI studies similarly demonstrate that improved accuracy may coexist with automation bias and overacceptance of algorithmic recommendations. Evidence of demographic performance disparities and limitations in the representativeness of training and validation datasets further raises concerns regarding fairness and equitable access to care. On this basis, we argue that cardiothoracic AI should be governed according to the level of decision influence rather than by technology type alone. We distinguish non-delegable professional duties, distributed system responsibilities, and non-transferable patient authority, and propose an Ethical Heart Team Framework for converting algorithmic output into ethically defensible clinical action.

## 1. Introduction

Artificial intelligence (AI) is now embedded in a widening range of cardiothoracic tasks, from preoperative risk estimation and imaging to operative planning, video analysis, complication prediction, and clinical decision support [1]. The growth of the field is measurable. Sulague et al. identified 81 AI studies in cardiac surgery through February 2024, with a marked acceleration after 2020. Random forest was used in 48 studies, support vector machines in 33, logistic regression in 32, and XGBoost in 31. The clinical distribution was similarly broad: 24.7% of studies involved valvular surgery, 11.8% coronary revascularization, 9.4% heart transplantation, 3.5% congenital cardiac surgery, and 2.4% aortic-dissection repair. Yet only 20 of the 81 studies examined patient outcomes, compared with 35 focused on model performance and 26 on clinician-related outcomes [2]. This imbalance is important: the literature is becoming technically mature faster than it is becoming clinically and ethically mature.

Cardiothoracic surgery is particularly sensitive to that gap because prediction is only one component of surgical judgment. Decisions about coronary revascularization, valve intervention, major pulmonary resection, transplantation, mechanical circulatory support, or extracorporeal membrane oxygenation (ECMO) require clinicians to combine estimated risk with anatomy, technical feasibility, timing, competing treatments, institutional capability, prognosis, patient goals, and uncertainty. A model can estimate the probability of an outcome; it cannot, by probability alone, determine whether an intervention is proportionate, whether a scarce resource should be allocated, or whether a particular trade-off is acceptable to the patient.

Recent specialty literature has therefore begun to move beyond the question of what AI can do and toward what its use does to surgical practice. Contemporary reviews in cardiothoracic and surgical journals address autonomy, bias, explainability, professional authority, and liability [3,4,5]. Magouliotis et al. [6] have explicitly framed surgical judgment as a central issue, while the EACTS-affiliated perspective by Engelhardt et al. [7] emphasizes the need for deliberate integration of AI within cardiothoracic practice. The unresolved question is no longer simply whether AI can outperform a conventional score or assist a technical task. It is whether, and under what conditions, that superior output should acquire authority over a clinical decision.

This review takes an evidence-led approach to that question. Rather than treating beneficence, non-maleficence, autonomy, justice, transparency, and accountability as a list of abstract principles, we examine the empirical situations in which these principles become difficult to protect. The central argument is that AI changes the ethical architecture of cardiothoracic surgery by redistributing epistemic authority: a clinician may be expected to endorse a recommendation generated by a system that they did not design, cannot fully interrogate, and may not be able to validate for the individual patient. The professional challenge is therefore to identify what can legitimately be delegated to algorithms and what must remain attributable to human clinical judgment, while also avoiding the opposite error of making surgeons solely responsible for failures rooted in software design, institutional deployment, or regulation.

## 2. Evidence Identification and Analytic Approach

The evidence base for this review was identified primarily through PubMed/MEDLINE and PubMed-indexed journal literature, with emphasis on publications from 2020 through 12 August 2026. Earlier studies were retained when they remained foundational to a specific clinical or ethical question. Searches combined terms related to artificial intelligence and machine learning with cardiothoracic and ethical concepts, including cardiothoracic surgery, cardiac surgery, thoracic surgery, risk prediction, clinical decision support, human-AI collaboration, automation bias, explainability, informed consent, fairness, transplantation, ECMO, liability, and surgical governance. Reference lists of recent cardiothoracic and surgical reviews were additionally examined to identify relevant primary studies. Official FDA and European Union sources were consulted for regulatory claims.

Studies were considered for inclusion when they addressed the development, validation, clinical application, human interaction, or ethical and governance implications of AI in cardiothoracic surgery, or when evidence from an adjacent medical domain directly informed an ethical issue for which cardiothoracic-specific evidence was limited. Particular attention was given to studies reporting quantitative model performance, external or multicentre validation, clinically relevant outcomes, clinician–AI interaction, demographic performance differences, patient perspectives, or responsibility and governance. Publications were excluded when they were not relevant to clinical decision-making or the ethical questions examined in this review, provided insufficient information to support the claim for which they were considered, or duplicated evidence more directly or comprehensively represented by another included source.

Study selection was performed according to relevance to the predefined thematic domains of the review: predictive performance and clinical utility; AI across the surgical pathway; human–AI collaboration and automation bias; bias and distributive justice; accountability, explainability, and informed consent; and regulation and lifecycle governance. Potentially relevant publications identified through database searching and reference-list review were assessed for their contribution to these domains, with preference given to the highest-quality and most directly applicable evidence. Because the purpose was to construct an evidence-informed ethical synthesis rather than to estimate a pooled treatment effect or comprehensively catalogue all published studies, formal duplicate independent screening and PRISMA flow-chart reporting were not undertaken. Throughout the synthesis, evidence derived directly from cardiac or thoracic surgical populations was distinguished from evidence extrapolated from adjacent medical fields. The latter was used only when cardiothoracic-specific empirical evidence was limited and is explicitly identified as such in the text; it was considered supportive rather than direct evidence for cardiothoracic practice.

Evidence was prioritized hierarchically: systematic reviews and meta-analyses; large national or multicentre cohorts; prospective or external-validation studies; empirical studies of clinicians or patients; and, where cardiothoracic evidence was sparse, high-quality evidence from adjacent medical domains. Quantitative results are reported when they materially change the ethical interpretation. Importantly, the absence of direct cardiothoracic evidence is treated as a knowledge gap rather than filled by assumption. Accordingly, this review should be interpreted as a structured, critical evidence synthesis rather than a PRISMA-compliant systematic review.

## 3. Predictive Performance: When Better Discrimination Is Not Yet Better Care

Risk prediction is the most mature cardiothoracic AI domain and therefore provides the clearest test of the difference between statistical performance and ethically justified clinical reliance. Benedetto et al. identified 459 citations and included 15 studies in a systematic review and meta-analysis comparing machine-learning models with logistic regression for operative mortality after cardiac surgery. When the best-performing machine-learning model from each study was pooled, the C-statistic was 0.88 (95% credibility interval, 0.83–0.93), compared with 0.81 (95% credibility interval, 0.77–0.85) for logistic regression (*p* = 0.03). The result established a real performance advantage at the level of discrimination. The authors nevertheless concluded that the magnitude and clinical influence of the improvement remained uncertain [8].

Three years later, Sinha et al. tested this question in 227,087 adults undergoing cardiac surgery in the United Kingdom, including 6258 in-hospital deaths (2.76%). XGBoost and random forest achieved AUCs of approximately 0.834 and 0.833–0.834, respectively, compared with 0.817–0.818 for EuroSCORE II. Machine-learning models showed lower calibration drift and greater net benefit on decision-curve analysis, but they did not significantly improve calibration overall. The investigators’ conclusion is ethically consequential: the statistical improvement was genuine, but its clinical impact was modest [9]. In other words, better discrimination did not automatically produce a sufficiently large change in decision quality to justify replacing established clinical reasoning [9,10].

The 2026 systematic review by Fuchs et al. extends the comparison. Thirteen studies published between 2020 and 2026 were included, with sample sizes ranging from 308 to 647,726. Across indications, machine-learning models were improved or comparable to conventional scores, with AUC differences ranging from 0.006 to 0.42. Some task-specific results were striking: XGBoost reached an AUC of 0.96 for postoperative infection, random forest reached 0.975 for major adverse events in type A dissection, and one study reported a net reclassification improvement of 0.550. Yet the review also identified limited external and temporal validation, concerns about overfitting and data leakage, and insufficient evaluation of fairness [11]. The trajectory from Benedetto to Sinha to Fuchs therefore shows increasing evidence of predictive capacity without equivalent evidence that the models are transportable, fair, or beneficial when embedded in real clinical workflows [8,9,11].

This distinction matters because risk scores can acquire more authority than their evidence warrants. A mortality probability is a descriptive estimate, not a treatment recommendation. A model predicting 20% perioperative mortality cannot determine whether the intervention is unacceptable without comparison with the risk of non-intervention, alternative treatment, reversibility of deterioration, the patient’s values, and the expected quality and duration of survival. The ethical problem is greatest when a risk score becomes an implicit veto: ‘high risk’ is transformed into ‘not a candidate’ without an explicit normative step [10,11].

The counterargument deserves equal weight. If a well-validated AI model consistently outperforms unaided judgment, ignoring it simply because the clinician prefers personal experience can itself violate beneficence and non-maleficence. Human primacy should therefore not mean that human intuition is automatically superior. The ethically defensible standard is calibrated reliance: the influence of a model should rise with evidence of external validity, calibration, subgroup performance, clinical utility, and fit to the current patient and institution. The principal quantitative studies underpinning these conclusions, together with their implications and the limits of what can reasonably be inferred from them, are summarized in Table 1 [8,9,10,11,12,13,14,15,16,17,18,19,20,21,22,23,24,25,26,27].

## 4. AI Across the Surgical Pathway: Useful Assistance, Uneven Evidence of Patient Benefit

The ethical significance of AI depends on where it sits in the surgical pathway. Technologies used for bounded tasks such as segmentation or planning are easier to validate and easier to override than systems that influence patient selection or life-sustaining treatment. Recent thoracic evidence illustrates both the clinical value of AI and the need to distinguish surrogate improvements from patient outcomes.

Chen et al. evaluated an AI-driven three-dimensional reconstruction system in a multicentre, multi-reader, multi-case crossover study. Ten thoracic surgeons assessed 140 cases with and without AI assistance. AI increased the accuracy of identifying anatomical variants by 8% and reduced related errors by 41%. Accuracy of operation-procedure selection improved by 8%, with a 35% reduction in selection errors; planning time fell by 25%, and user satisfaction reached 99%. These are meaningful clinical-process outcomes because anatomic misunderstanding can alter segmentectomy planning. They also demonstrate why a blanket anti-AI position is untenable: in a bounded task with clear reference standards, AI can make the surgeon’s preparation more accurate and efficient [12].

However, the next stage of evidence is more instructive. Geng et al. retrospectively studied 1197 patients scheduled for segmentectomy (*n* = 479) or lobectomy (*n* = 718). After propensity-score matching, planned and actual procedures were consistent in 97.3% of AI-3D-assisted segmentectomies versus 80.0% with two-dimensional CT alone (*p* < 0.001) [13]. Yet intraoperative and postoperative outcomes were otherwise largely comparable. The comparison between Chen and Geng is important: better information can improve planning fidelity without necessarily producing detectable improvements in blood loss, conversion, reoperation, drainage, or other hard outcomes [12,13]. The ethical conclusion should therefore be proportionate to the evidence. AI-3D appears useful; claims that it improves patient outcomes more broadly remain premature.

Intraoperative computer vision is advancing in parallel. Liang et al. developed LungSurg using 222 video-assisted thoracoscopic lobectomy videos collected prospectively from eight centres, with more than 32,000 annotations and over one million frames containing phase information. On external validation, mean average precision for segmentation was 0.745 for the left lung and 0.726 for the right lung. The phase-classification network achieved 71.5% Top-1 and 88.0% Top-3 accuracy across 14 surgical phases. LungSurg performed comparably with senior surgeons for anatomical identification and showed higher sensitivity, while residents exposed to the system improved in educational tasks [14]. This is strong evidence of technical feasibility, but it is not evidence for autonomous operative judgment. As AI moves from displaying anatomy to recommending or executing action, the key safety metric becomes recoverability: whether the human operator can recognize failure, interrupt the system, and rescue the patient before an error becomes irreversible [14,15].

Postoperative prediction creates a different ethical problem. Kalisnik et al. analysed 7507 cardiac-surgery patients, of whom 1699 (22.6%) developed acute kidney injury (AKI). Their Detect-A(K)I model identified AKI within 12 h with an AUC of 0.88, sensitivity of 78.0%, specificity of 78.9%, and overall accuracy of 82.1%. In this context, an early signal can prompt confirmatory assessment and closer monitoring, making AI a plausible form of augmentation. By contrast, when prognostic models are used for mortality or ECMO outcomes, the downstream action can itself affect the outcome. If predicted futility leads clinicians to reduce treatment intensity, prediction can contribute to a self-fulfilling prophecy. For that reason, mortality probability should inform—but not independently determine—withdrawal or limitation of life-sustaining therapy [16].

Taken together, the pathway evidence suggests a gradient of ethical risk. AI used to organize or reveal information can often be validated against a bounded reference standard. AI used to recommend treatment crosses from technical assistance into normative influence. AI used to determine access to surgery, transplantation, ECMO, or withdrawal of treatment crosses further still, because the output becomes connected to distributive justice, patient values, and potentially irreversible consequences.

**Figure 1:**
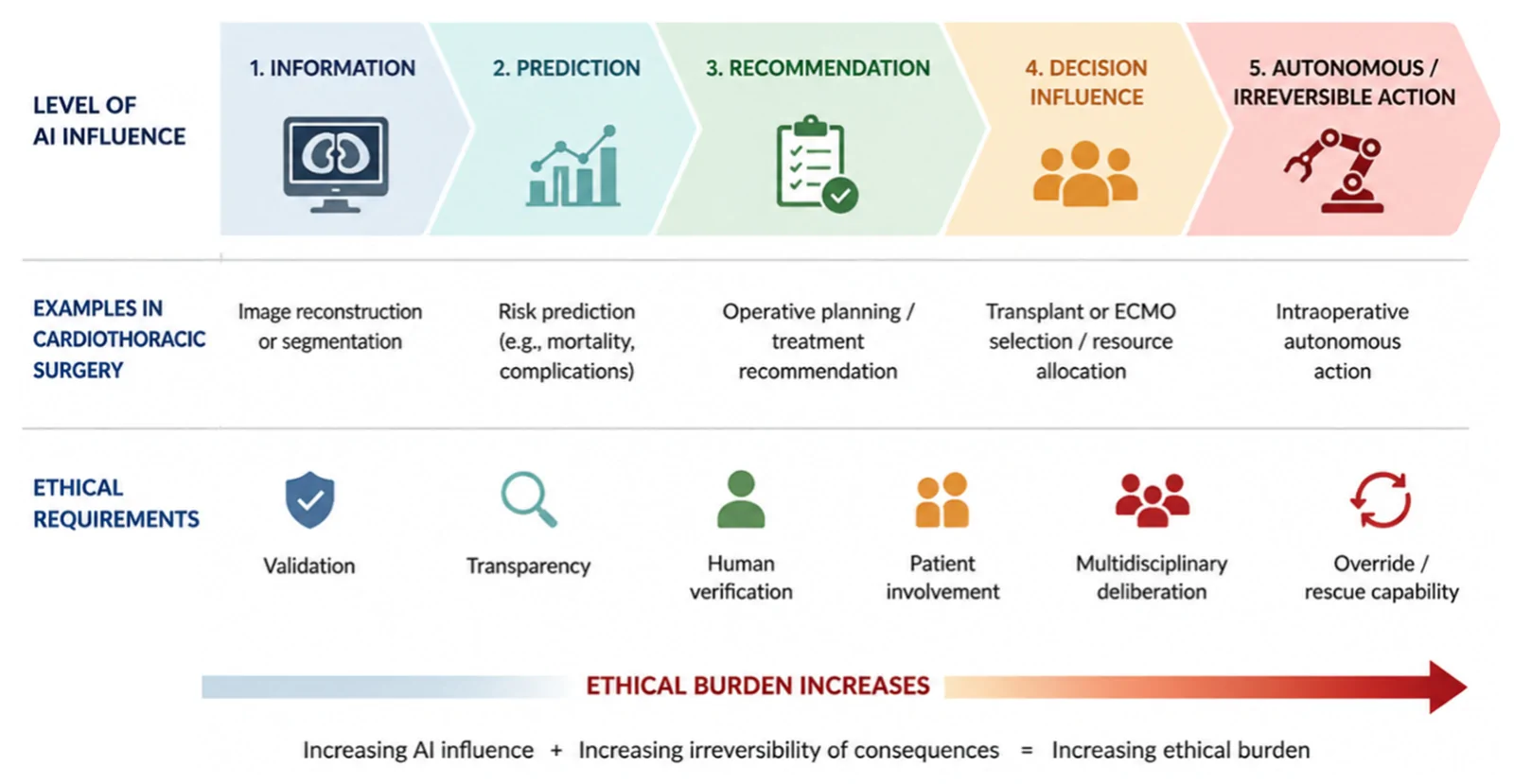
From augmentation to delegation: the ethical risk gradient of artificial intelligence in cardiothoracic surgery. The ethical significance of AI increases as its role progresses from information processing and risk prediction toward treatment recommendation, high-level decision influence, and potentially autonomous or irreversible action. Illustrative cardiothoracic applications are shown along this continuum. Increasing algorithmic influence and irreversibility of clinical consequences are associated with a progressively greater ethical burden and a corresponding need for human oversight and accountability.

A distinction is therefore required between AI as decision support and AI as a source of effective decision influence. In a strictly supportive role, the system organizes information, estimates risk, identifies patterns, or presents options while leaving the clinician’s independent assessment and the available choice set substantially unchanged. By contrast, AI acquires effective decision influence when its output materially shapes which options are considered, how they are ranked, whether a patient is regarded as eligible for an intervention, or which course of action is ultimately preferred. This distinction depends less on whether the system formally ‘makes’ the decision than on its actual influence within the clinical workflow. A nominally advisory recommendation may therefore carry substantial ethical weight when clinicians routinely defer to it, when it determines access to treatment, or when institutional processes make deviation difficult. Conversely, technically sophisticated AI may remain ethically closer to augmentation when its output is genuinely contestable and clinicians retain meaningful independent judgment. This progression from decision support toward effective decision influence can be conceptualized as an ethical risk gradient, in which increasing AI influence and increasing irreversibility of the resulting clinical consequences are accompanied by a progressively greater ethical burden (Figure 1).

## 5. Human–AI Collaboration: Accuracy Gains, Automation Bias, and the Preservation of Surgical Judgment

The ethical performance of clinical AI cannot be inferred from model performance alone because decisions are made by human–system teams. The relevant unit of analysis is therefore not only the algorithm, but the interaction between the algorithm and the clinician. The first direct cardiothoracic evidence on this interaction is already cautionary [17].

Leon et al. constructed 15 high-fidelity complex cardiac-surgery scenarios and evaluated five large language models using a ten-dimensional framework developed by senior cardiac surgeons. Median normalized scores ranged from 0.521 for Llama3-OpenBioLLM-70B to 0.896 for O1. Yet aggregate competence concealed important weaknesses: scenario comprehension scored 0.920, whereas patient safety scored only 0.507, hallucination avoidance 0.549, and clinical efficiency 0.597. In the second blinded evaluation phase, surgeons were shown expert reference answers and could revise their initial judgments. Across models, 7.57% of ratings changed from affirmative to negative, compared with only 2.59% from negative to affirmative; among the five highest-weighted domains, 10.16% of ratings were revised from affirmative to negative. The authors identified overacceptance of plausible but incorrect reasoning as the dominant collaboration imbalance [18].

Experimental evidence outside cardiothoracic surgery shows the same mechanism in a more controlled form. Wang et al. asked 40 clinicians to evaluate 200 anterior cruciate ligament cases with and without AI support. AI improved mean diagnostic accuracy from 87.2% (SD 13.1%) to 96.4% (SD 1.9%; *p* < 0.001), demonstrating a clear benefit. At the same time, automation bias accounted for 45.5% of errors remaining in the AI-assisted condition and affected clinicians across levels of expertise. The authors estimated that suppressing AI outputs with a high probability of misleading the clinician could reduce automation bias by 41.7% [19]. This is a particularly useful comparison for surgery: the same technology can simultaneously increase aggregate accuracy and create a new, predictable class of human error.

The appropriate professional response is therefore neither unconditional trust nor algorithm aversion, it is calibrated trust [20,21]. The more reliable and externally validated the model, the more weight its output may deserve; the more consequential, unfamiliar, or poorly validated the application, the more active the human challenge should become [21]. This also means that a surgeon who repeatedly ignores a strongly validated system without a defensible reason may act as irresponsibly as one who follows it uncritically.

Surgeons appear to recognize both the opportunities and the governance problem. In the international cardiothoracic survey reported by Platz et al., 424 complete responses showed heterogeneous views but broad support for proactive specialty involvement. A related analysis of 412 respondents found that 59% anticipated improved operative outcomes and 63% expected lower error rates with AI; enthusiasm was strongest for administrative uses such as billing and coding (84%), note writing (69%), quality improvement (64%), and outpatient testing (59%). At the same time, 67% supported external regulation of clinical AI, 80% supported partnership between cardiothoracic societies and industry, 81% supported sharing educational content, and 69% supported access to specialty clinical databases for development [22,23]. These findings do not show professional consensus on the proper role of AI, but they do show that surgeons distinguish relatively low-risk automation from clinically influential applications.

Training must reflect the same distinction. AI literacy should not be reduced to learning how to operate a system. It should include knowledge of intended use, external validation, calibration, uncertainty, subgroup performance, version changes, and failure modes [24]. Just as importantly, training should preserve unaided reasoning and technical rescue skills. If trainees encounter algorithmic answers before they have formed an independent assessment, there is a plausible risk that they learn to supervise recommendations without developing the competence required to challenge them. Direct longitudinal cardiothoracic evidence for deskilling is still limited; it should be treated as a research priority rather than a demonstrated outcome.

## 6. Bias, Justice, and the Risk of Allocation by Prediction

Fairness concerns in cardiothoracic AI are no longer hypothetical. Although derived from cardiovascular imaging rather than a cardiothoracic surgical population, Puyol-Antón et al. evaluated automated cardiac magnetic resonance segmentation in 5903 UK Biobank participants (52% male, 81% White). Overall performance was high, but Dice scores differed substantially by race: approximately 94% in White participants versus 86–89% in minority ethnic groups. The disparity persisted after adjustment for potential confounders, and race remained the main factor explaining the difference. A model can therefore appear highly accurate at population level while performing materially worse for specific groups [25].

Straw et al. demonstrated a related problem for sex. Their literature search identified 127 cardiac machine-learning papers; 60 met criteria for full review, but only three explicitly highlighted sex differences in performance, and women were consistently underrepresented where sex was reported. When the authors reproduced published algorithms on two open cardiac datasets, random-forest accuracy was approximately 84.2% and 85.7%, yet women had a significantly higher false-negative rate in 13 of 16 experiments in one dataset [26]. The clinical meaning is direct: underprediction of disease in women can reduce access to further investigation or treatment even when average model accuracy appears acceptable.

Transplantation intensifies the ethical consequences because prediction may influence access to a scarce, life-saving resource. Sargiotis et al.screened 122 studies and included 15 studies of AI prediction after heart or lung transplantation. Reported AUCs ranged from 0.620 to 0.921, and random forest and XGBoost often outperformed traditional linear models. However, North American White populations predominated, paediatric populations were absent, and most studies were judged at high overall risk of bias [27]. These limitations do not negate the predictive value of the models; they make it unsafe to convert prediction directly into allocation authority.

The most important ethical distinction is between prediction and entitlement. A model may estimate that one patient has a lower probability of survival than another, but the conclusion that the first patient should not receive treatment is not contained in the probability. It requires decisions about acceptable benefit, scarcity, proportionality, disability, duration and quality of survival, opportunity cost, and the legitimacy of the threshold being used. If those value judgments are embedded invisibly in an algorithmic cut-off, distributive decisions become harder to contest [28].

We describe this risk as allocation amplification: a modest performance disparity can become a large inequity in access when an algorithmic score functions as a gatekeeper. The ethical importance of a fairness defect therefore depends on its downstream use. A subgroup difference in automated segmentation and the same subgroup difference in a transplant-listing model are not ethically equivalent. High-stakes systems require subgroup-specific validation and auditing of downstream decisions, not only global performance metrics.

Fairness also cannot be guaranteed by deleting protected variables such as sex or race. Demographic information may remain encoded in correlated clinical variables, referral patterns, socioeconomic exposures, labels, and missingness. The practical requirement is continuous evaluation of who is represented, how the model behaves in relevant subgroups, and whether its use changes access to treatment.

## 7. Accountability, Explainability, and Informed Consent

When AI contributes to a harmful surgical decision, the question ‘Who is responsible?’ has no credible one-person answer. Hashimoto et al., in the SAGES white paper on AI risk and liability in surgery, proposed a tripartite framework: risks inherent to the AI system, risks introduced by the clinician-user, and risks arising from institutional deployment. This structure is more realistic than assigning every adverse event to the surgeon. A model may fail because it was poorly designed, trained on unrepresentative data, updated without adequate validation, implemented outside its intended use, presented through a misleading interface, or relied on uncritically by a clinician [29]. Those are different failures and they imply different responsibilities.

Empirical work supports this distributed view. Duffourc et al. conducted six focus groups with 18 surgeons (11 in the European Union and seven in the United States). Participants accepted that surgeons retain responsibility when using AI, but also emphasized governance, the possibility that AI may change the standard of care, uncertainty around manufacturer liability, and the importance of patient information and consent [30]. Broader survey evidence also shows that responsibility is perceived as shared rather than singular. Khullar et al. found that both the US public and physicians most often assigned responsibility to the clinician when an AI-assisted error occurred, although the public did so more frequently (66.0% vs. 57.3%; *p* = 0.020). Physicians were more likely than the public to assign liability to vendors (43.8% vs. 32.9%; *p* = 0.004) and healthcare organizations (29.2% vs. 22.6%; *p* = 0.05). The pattern mirrors the normative problem: clinical accountability remains, but system responsibility does not disappear [31].

**Figure 2:**
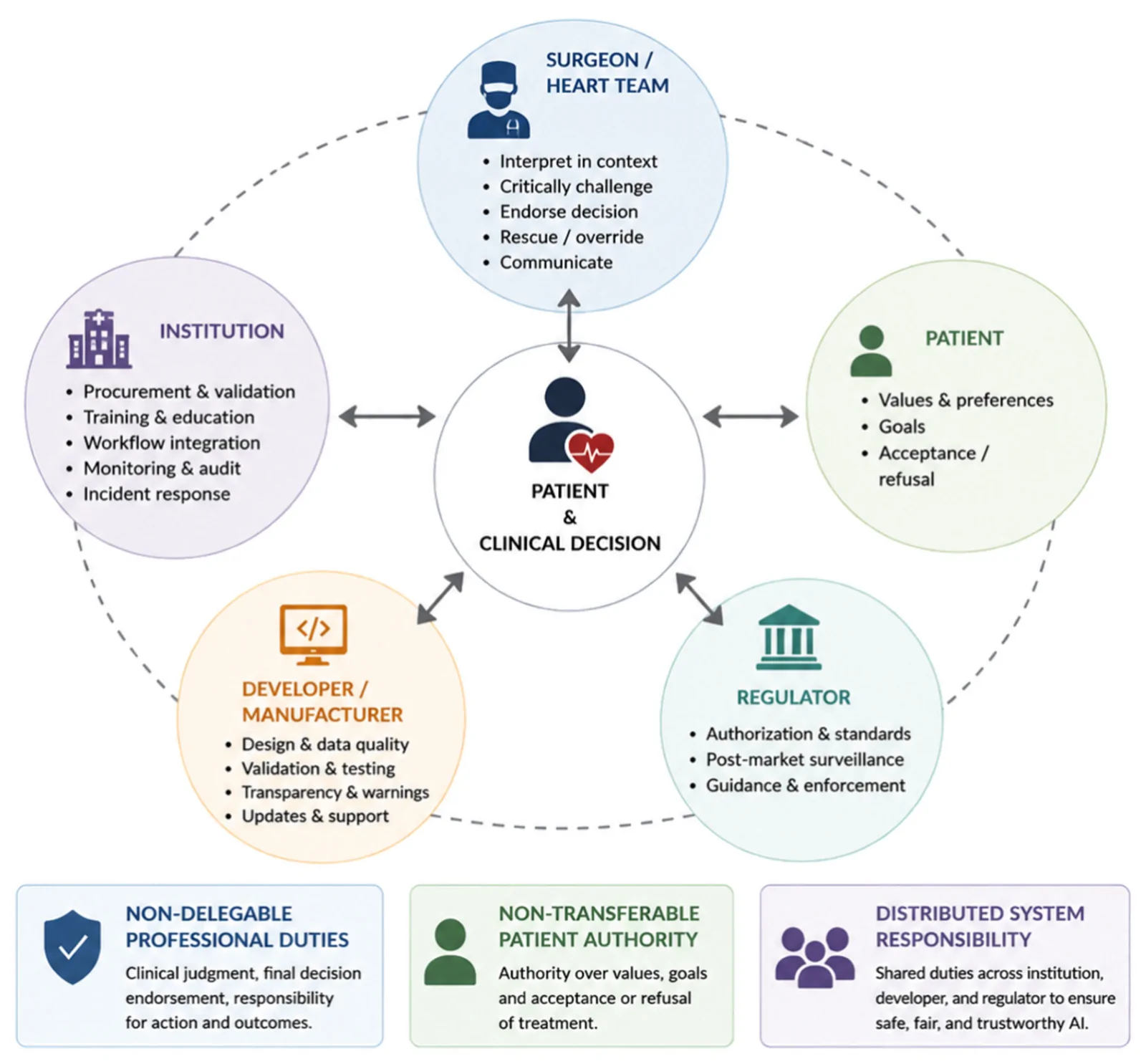
Distributed accountability and non-delegable authority in AI-assisted cardiothoracic surgery. AI contributes to clinical decision-making within a network of distinct but interconnected responsibilities. Surgeons and Heart Teams retain non-delegable professional duties related to contextual interpretation, critical appraisal, clinical endorsement, communication, and rescue or override. Patients retain non-transferable authority over their values, goals, and acceptance or refusal of treatment. Institutions, developers and manufacturers, and regulators carry complementary responsibilities for implementation, validation, monitoring, system design, updates, authorization, and surveillance. AI may inform the clinical decision but does not itself become an accountable moral or professional agent.

A useful distinction is therefore between non-delegable professional responsibility and distributed system responsibility. The surgeon or clinical team remains responsible for deciding whether an output is applicable, integrating it with the patient’s clinical context, recognizing discordant or unsafe advice, communicating the reasoning, and accepting or rejecting the recommendation. Developers and manufacturers remain responsible for design, validation, intended-use claims, warnings, and controlled updates; institutions for procurement, local validation, credentialing, monitoring, version control, and incident response; regulators for authorization and surveillance. This model prevents two opposite errors: ‘the AI told me to’ as a defence of poor clinical judgment, and the use of the surgeon as a liability sink for failures outside meaningful clinical control. Accordingly, accountability in AI-assisted cardiothoracic surgery should be understood as a distributed structure in which professional duties remain non-delegable, patient authority over values and treatment choices remains non-transferable, and system-level responsibilities are shared among institutions, developers, manufacturers, and regulators (Figure 2).

Explainability should be understood within the same framework. Complete interpretability is neither technically possible for every model nor automatically useful. Amann et al. argue that the value of explanation depends on technical feasibility, context, the role assigned to the system, and the needs of the user [32]. The relevant clinical question is therefore not whether every parameter can be explained, but whether the professional has sufficient epistemic access to use the output responsibly. At minimum, high-stakes users should know the intended use, development and validation population, key inputs, outcome definition, uncertainty, known failure modes, subgroup limitations, and whether the current patient is outside the validated domain. We refer to this as risk-proportionate epistemic transparency [32,33].

Consent adds the patient’s perspective to this accountability structure. The use of AI does not automatically require a separate consent process for every background function, but empirical evidence from broader medical settings indicates that patients can consider AI involvement material. Park studied 1000 respondents and found that information about AI use in diagnosis was perceived as more important when AI actually participated in the physician’s decision. Preferences varied significantly by age, sex, and income, arguing against a one-size-fits-all disclosure script [34]. Rose and Shapiro similarly propose that notification and consent should depend on factors such as model autonomy, departure from usual practice, whether the system is patient-facing, and the clinical risk introduced by the model [35].

Thoracic surgery offers a concrete patient-information example. Ferrari-Light et al. presented ChatGPT responses to 30 common lung-cancer-surgery questions to nine thoracic surgeons from four academic institutions. Mean quality scores ranged from 3.1 to 4.2 on a five-point scale. Minor inaccuracies were identified by at least one surgeon in 100% of responses and major inaccuracies in 36.6%. Although 66.7% of surgeons considered ChatGPT-5.6 an accurate information source, only 55.6% regarded the answers as comparable with those of experienced thoracic surgeons and only 44.4% would recommend the tool to patients [36]. The data support augmentation rather than substitution: AI-generated education may be useful, but professional verification remains necessary when information forms part of informed consent [37].

For cardiothoracic care, disclosure should therefore be linked to influence. AI that silently improves image reconstruction does not have the same ethical significance as AI that materially changes a recommendation for CABG versus PCI, SAVR versus TAVI, resection strategy, transplantation, or continuation of ECMO. As algorithmic influence increases, so should the obligation to explain that influence, its uncertainty, the role of human review, and the availability of alternatives [38]. These distinctions translate into a practical allocation of responsibilities across the different actors involved in AI-assisted cardiothoracic care, while identifying those clinical and patient-related functions that should not be delegated to AI (Table 2).

## 8. The Ethical Heart Team Framework: From Algorithmic Output to Defensible Clinical Action

### 8.1. Structure and Operational Principles of the Ethical Heart Team Framework

The evidence reviewed above suggests that cardiothoracic AI should not be governed by a generic rule such as ‘the surgeon must always overrule the machine’ or ‘the most accurate model should decide.’ Both are too crude. The appropriate level of human scrutiny depends on the clinical consequence of the output, the strength of validation, the possibility of detecting error, the degree of uncertainty, and the extent to which the decision incorporates patient values or scarce-resource allocation [39].

We therefore propose an Ethical Heart Team Framework organized around four sequential gates. The framework is deliberately practical and evidence-linked rather than a new set of abstract ethical principles.

Gate 1: Applicability. Before an AI output is allowed to influence a decision, the team asks whether the system is valid for this patient and setting. The recurring external-validation limitations described by Fuchs et al. [11], the calibration drift observed by Sinha et al. [9], and the demographic performance gaps identified by Puyol-Antón et al. demonstrate why model performance cannot simply be transported across populations [25]. A high internal AUC is not evidence of universal applicability.

Gate 2: Epistemic sufficiency. The team asks whether it understands enough about the output to rely on it responsibly. This is not a demand for complete mathematical transparency. It requires sufficient knowledge of intended use, uncertainty, validation, limitations, and failure modes to determine whether acceptance is reasonable. The more irreversible the decision, the higher this requirement should be.

Gate 3: Equity and downstream consequence. The team asks whether subgroup bias or dataset limitations could alter treatment access. This gate is especially important in high-risk surgery, transplantation, ECMO, and prognosis-based treatment limitation. Where subgroup performance is unknown, an algorithm should not function as an automatic exclusion threshold.

Gate 4: Human deliberation and patient alignment. The AI output is compared with the full clinical picture, multidisciplinary judgment, alternative treatments, and patient goals. Discordance should trigger explicit discussion rather than automatic deference to either clinician or algorithm. The final action is then accepted, modified, or rejected through accountable professional endorsement. When the final clinical decision diverges materially from the AI-generated recommendation, the discrepancy and the rationale for the clinician’s decision should be documented in the patient record. Such documentation supports clinical accountability and continuity of care, provides a transparent record of the decision-making process, and facilitates subsequent communication with the patient.

**Figure 3:**
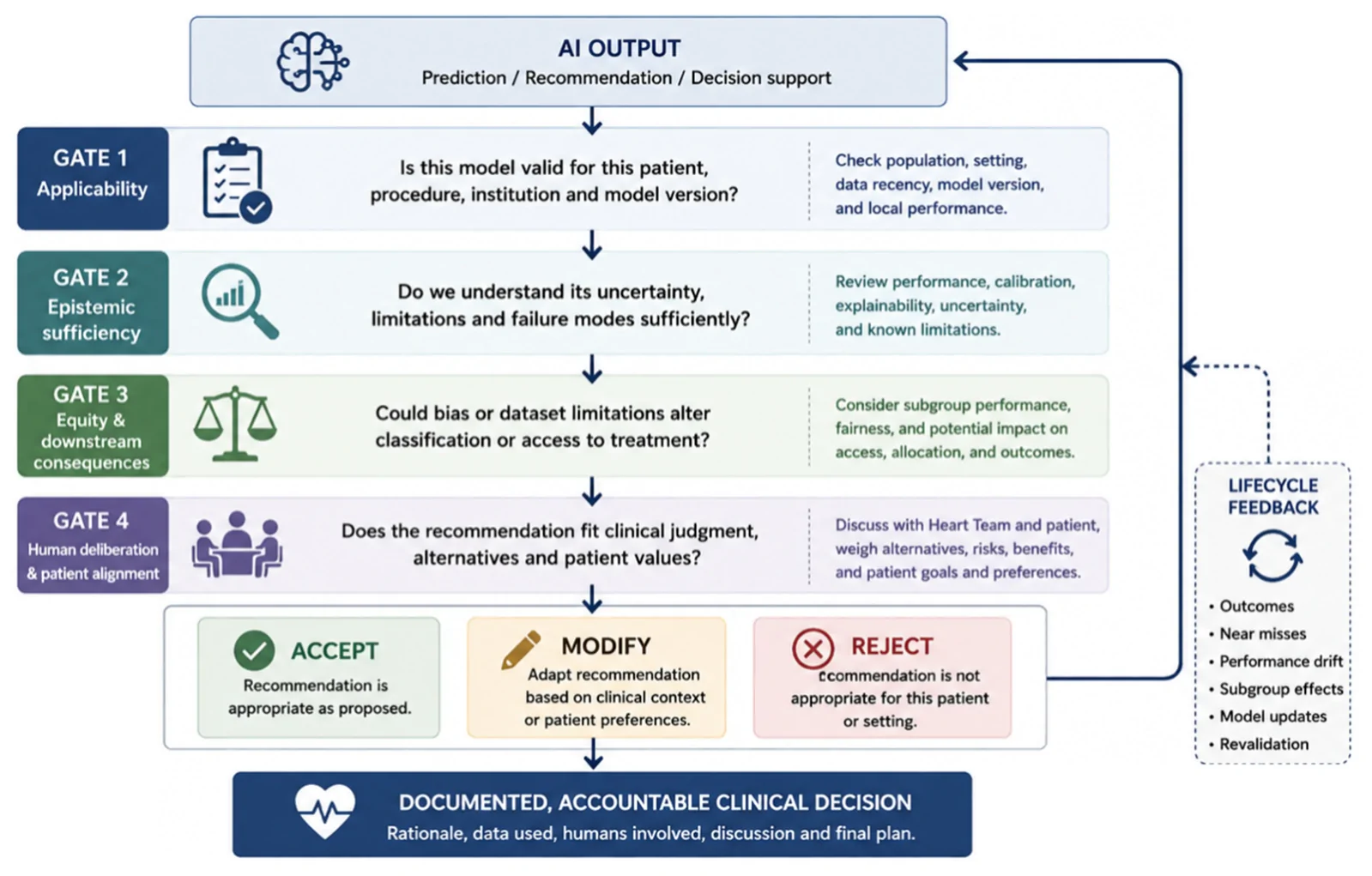
The Ethical Heart Team Framework for translating AI-generated output into ethically defensible clinical decisions.

Together, these four gates form a sequential but iterative pathway through which AI-generated output is evaluated, contextualized, and ultimately accepted, modified, or rejected through accountable clinical judgment, with post-deployment experience feeding back into subsequent assessment (Figure 3).

The framework separates three forms of authority. First, non-delegable professional duties include contextual interpretation, critical challenge, recognition of failure, rescue or override, documentation, and communication. Second, distributed system responsibilities include data governance, validation, cybersecurity, version control, local implementation, and post-deployment surveillance. Third, patient authority over values, goals of care, and acceptance or refusal of treatment remains non-transferable. This distinction is more precise than ‘human primacy’ because it preserves patient autonomy and acknowledges that institutions and manufacturers cannot transfer all responsibility to the end user.

The empirical studies also suggest a risk-proportionate rule for implementation. Low-consequence automation may require routine validation and monitoring. Intermediate decision support requires external or strong local validation, uncertainty display, subgroup assessment, and clinician confirmation. High-consequence AI influencing treatment selection or scarce resources requires explicit multidisciplinary deliberation, auditability, and a documented override pathway. Intraoperative or semi-autonomous systems capable of irreversible action require the highest standard: prospective safety evidence, immediate human override, rescue capability, version traceability, and continuous surveillance. Importantly, classification along this continuum should reflect the system’s actual influence on clinical decisions rather than its formal designation as ‘decision support’; an advisory system that routinely determines treatment ranking, eligibility, or clinician choice should be governed according to that higher level of effective influence. The resulting framework translates these principles into a structured set of decision gates, linking each ethical question to the relevant evidence trigger and the action required when concerns arise (Table 3).

### 8.2. Clinical Application of the Ethical Heart Team Framework

A hypothetical transplant-selection scenario illustrates how the framework may operate in practice. Consider a patient with advanced heart failure undergoing evaluation for heart transplantation, for whom an AI-based model predicts a high risk of post-transplant mortality. At Gate 1 (applicability), the Heart Team should determine whether the model has been adequately validated in patients comparable to the individual under consideration and whether its intended use includes transplant-candidate assessment. At Gate 2 (epistemic sufficiency), the team should examine the reliability and uncertainty of the prediction and identify the clinical variables driving the high-risk classification rather than treating the output as a definitive prognosis. At Gate 3 (equity and downstream consequence), subgroup performance and the consequences of a false-positive high-risk classification require particular scrutiny because algorithmic overestimation of risk could contribute to exclusion from a scarce, potentially life-saving resource. At Gate 4 (human deliberation and patient alignment), the prediction should be integrated with the complete clinical assessment, alternative treatment options, multidisciplinary judgment, and the patient’s goals and preferences. The AI output may therefore inform, but should not independently determine, transplant eligibility; any material divergence between the algorithmic recommendation and the final clinical decision should be explicitly discussed and documented in the patient record. The same sequence can be applied to other high-consequence cardiothoracic decisions, including ECMO candidacy and AI-assisted selection of operative strategy, with the intensity of scrutiny increasing according to the reversibility and clinical consequences of the decision.

## 9. Regulation and Lifecycle Governance

Recent regulation is moving in the same direction as the evidence: toward risk management, human oversight, and lifecycle accountability rather than one-time technical approval. Regulation (EU) 2024/1689, the EU Artificial Intelligence Act, establishes risk-management, data-governance, transparency, accuracy, robustness, cybersecurity, and human-oversight obligations for relevant high-risk systems, including certain AI associated with regulated medical products [40]. The emphasis on effective oversight is important because the human role must be operational, not merely formal.

In the United States, the FDA’s 2026 Clinical Decision Support Software guidance emphasizes, for relevant non-device CDS functions, that health professionals should be able to independently review the basis of a recommendation rather than rely primarily on it [41]. The FDA’s 2025 final guidance on Predetermined Change Control Plans for AI-enabled device software functions also requires planned modifications, validation methodology, and impact assessment to be described in advance [42]. For cardiothoracic departments, this makes version control an ethical as well as technical issue: the evidence supporting one model version cannot automatically be assumed to support a modified one.

Governance should therefore begin before deployment and continue after it. Hospitals need processes for procurement, local validation, clinician training, documentation of intended use, monitoring of subgroup performance, incident reporting, drift detection, and temporary suspension when safety signals emerge. Specialty societies have an important role in defining acceptable evidence thresholds and competencies, a role that cardiothoracic surgeons themselves support in contemporary survey data [22,23,43,44].

## 10. Limitations and Research Priorities

The evidence base remains uneven. Cardiac-surgery risk prediction is supported by meta-analysis and very large cohorts, whereas direct evidence on Heart Team behavior, long-term deskilling, informed consent, and AI-driven allocation decisions is limited. Several ethical claims in this review therefore represent evidence-informed normative synthesis rather than effects proven in randomized cardiothoracic trials. Evidence from adjacent fields, such as the controlled automation-bias study by Wang et al., is useful but should not be mistaken for direct cardiac-surgery evidence [19]. Accordingly, findings extrapolated from non-cardiothoracic settings should be interpreted as mechanistic or conceptual support and require specialty-specific validation before they are translated into cardiothoracic clinical practice.

The proposed Ethical Heart Team Framework is conceptual and has not yet undergone prospective clinical validation; its feasibility, reproducibility, and effect on decision quality should therefore be evaluated in future cardiothoracic studies.

The next generation of studies should measure what happens after AI enters the workflow. Priorities include prospective human–AI decision studies in Heart Teams; trials comparing interface designs, uncertainty displays, and deliberate suppression of unreliable outputs; subgroup-specific consequences of risk models; registries of AI-related adverse events and near misses; longitudinal assessment of trainee competence; and patient studies testing when AI involvement becomes material to consent. Performance metrics should be accompanied by calibration, external validation, fairness analyses, and decision-impact outcomes rather than AUC alone.

## 11. Conclusions

The current evidence does not support either technological enthusiasm or technological rejection as a sufficient ethical position. Machine learning can improve risk discrimination after cardiac surgery, AI-assisted reconstruction can improve thoracic planning, computer vision can identify operative anatomy and phases, and predictive systems can detect postoperative complications earlier. At the same time, the size of statistical gains is often larger than the demonstrated gain in patient outcomes, external validation remains inconsistent, subgroup disparities are measurable, and human–AI collaboration can generate automation bias and overacceptance.

The ethical challenge is therefore a problem of justified influence. The more an AI system shapes an irreversible, preference-sensitive, or distributive decision, the stronger the requirements for validation, epistemic transparency, fairness assessment, human challenge, patient communication, and lifecycle monitoring. AI may legitimately perform calculations, reveal patterns, and generate recommendations; it should not become the unaccountable bearer of surgical judgment. Cardiothoracic surgery should preserve non-delegable professional duties without pretending that responsibility is exclusively individual. Contextual interpretation, accountable endorsement, communication, failure recognition, and rescue remain professional obligations. Model design, validation, deployment, version control, and surveillance are distributed responsibilities. Patient values remain the patient’s authority. The Ethical Heart Team Framework translates these distinctions into a practical sequence for deciding when algorithmic assistance remains augmentation and when it risks becoming an ethically unacceptable delegation of judgment.

## Figures and Tables

**Table 1:** Comparative quantitative evidence relevant to ethical decision-making in cardiothoracic AI.

Study	Design/Sample	Key Quantitative Finding	What the Result Supports	What it Does not Establish
Benedetto et al., 2022 [8]	Meta-analysis; 15 studies	Pooled C-statistic 0.88 for ML vs. 0.81 for logistic regression; *p* = 0.03	ML can improve mortality discrimination	Clinical benefit or safe replacement of judgment
Sinha et al., 2023 [9]	UK national cohort; *n* = 227,087; deaths = 6258 (2.76%)	AUC ~0.834 (XGBoost) vs. 0.817–0.818 (EuroSCORE II); modest clinical impact	Large-scale comparative performance; calibration drift	Large patient-level outcome benefit from ML
Fuchs et al., 2026 [11]	Systematic review; 13 studies; *n* = 308–647,726	AUC gains 0.006–0.42; NRI 0.550 in one study; external validation limited	Performance gains across tasks; persistent methodological weaknesses	Generalizability or fairness across settings
Chen et al., 2025 [12]	Multicentre crossover; 10 thoracic surgeons, 140 cases	+8% anatomic-variant accuracy; 41% fewer errors; 25% less planning time; 99% satisfaction	AI-3D can improve planning performance	Improved hard postoperative outcomes
Geng et al., 2026 [13]	Retrospective; *n* = 1197; propensity matching	Segmentectomy plan-procedure consistency 97.3% vs. 80.0%; *p* < 0.001; other outcomes comparable	Greater fidelity to preoperative planning	Broad perioperative outcome superiority
Kalisnik et al., 2022 [16]	Cardiac surgery cohort; *n* = 7507	AKI 22.6%; AUC 0.88; sensitivity 78.0%; specificity 78.9%; accuracy 82.1%	Early postoperative risk detection	That prediction should determine treatment escalation/limitation
Leon et al., 2026 [18]	15 complex cardiac scenarios; 5 LLMs; blinded surgeon evaluation	Best model score 0.896; patient-safety dimension 0.507; 7.57% ratings revised positive-to-negative	Capability plus measurable over acceptance risk	Safe autonomous use in complex cardiac decisions
Puyol-Antón et al., 2022 [25]	UK Biobank CMR; *n* = 5903	Dice ~94% White vs. 86–89% minority ethnic groups	Demographic performance disparity can exist despite high overall accuracy	Direct proof of treatment-access discrimination
Straw et al., 2024 [26]	127 papers screened; 60 reviewed; replicated cardiac ML experiments	Only 3 papers examined sex differences; female FNR higher in 13/16 experiments	Under-recognized sex bias in cardiac ML	That every cardiac AI model is biased
Sargiotis et al., 2024 [27]	Systematic review; 15/122 studies included	AUC 0.620–0.921; predominantly North American/White samples; pediatric cohorts absent; high risk of bias common	Predictive promise with major representativeness concerns in transplant	Safe algorithmic allocation of scarce organs

**Table 2:** Responsibility in AI-assisted cardiothoracic care.

Domain	Primary Accountable Actor(s)	Evidence-Informed Requirement	Delegable to AI?
Clinical applicability	Surgeon/Heart Team	Check intended use, population, calibration, patient-specific fit	No
Professional endorsement	Treating clinician/Heart Team	Accept, modify, or reject with reasoned justification	No
Patient values and choice	Patient/representative	Goals, trade-offs, acceptance or refusal of treatment	No
Failure recognition/rescue	Clinical team	Preserve override authority and competence when system fails	No
Model design/validation	Developer/manufacturer	Representative data, performance evidence, warnings, update control	Not a clinician-delegable duty
Local implementation	Institution	Procurement, local validation, credentialing, workflow design, monitoring	Shared system responsibility
Lifecycle surveillance	Institution + developer + regulator	Version tracking, drift, incident reporting, corrective action	Automation may assist; accountability remains human/institutional

**Table 3:** Ethical Heart Team Framework: evidence-linked decision gates.

Gate	Question Before Relying on AI	Evidence Trigger	Required Action if Concern Exists
1. Applicability	Was the model validated for this patient, institution, procedure, and version?	External-validation limits; calibration drift; dataset shift	Downgrade AI influence; obtain alternative evidence; local review
2. Epistemic sufficiency	Are uncertainty, limitations, and the basis of the recommendation sufficiently understood?	Black-box limitations; cognitive burden; changing model versions	Increase independent review; seek interpretable evidence; do not rely primarily on output
3. Equity	Could subgroup performance or dataset representation alter access or risk classification?	Race/sex performance gaps; transplant representativeness limits	Subgroup audit; independent reassessment; prohibit automatic exclusion threshold
4. Human deliberation & patient alignment	Does the output fit clinical context, Heart Team judgment, alternatives, and patient values?	Automation bias; overacceptance; preference-sensitive choices	Discuss discordance explicitly; accept/modify/reject with rationale documented in the patient record
Lifecycle feedback	Did outcomes, near misses, drift, or subgroup effects change after deployment?	Post-market performance and model updates	Incident reporting; recalibration; retraining/revalidation; suspension if necessary

## Data Availability

No new data were created or analyzed in this study. Data sharing is not applicable to this article.

## References

[1] Khodaveisi T., Aslani N., Amiri P., Kamrani F., Saeedi S. (2025). Application of artificial intelligence in predicting the results of open-heart surgery: A scoping review. BMC Med. Inform. Decis. Mak..

[2] Sulague R.M., Beloy F.J., Medina J.R., Mortalla E.D., Cartojano T.D., Macapagal S., Kpodonu J. (2024). Artificial intelligence in cardiac surgery: A systematic review. World J. Surg..

[3] Arjomandi Rad A., Vardanyan R., Athanasiou T., Maessen J., Sardari Nia P. (2025). The ethical considerations of integrating artificial intelligence into surgery: A review. Interdiscip. Cardiovasc. Thorac. Surg..

[4] Wah J.N.K. (2025). Revolutionizing surgery: AI and robotics for precision, risk reduction, and innovation. J. Robot. Surg..

[5] Rashidi P., Kilic A., Kline A., Liu T., McCarthy P.M., Johnston D.R., Sade R.M. (2025). Artificial intelligence and machine learning in cardiothoracic surgery: Future prospects and ethical issues. J. Thorac. Cardiovasc. Surg..

[6] Magouliotis D.E., Sicouri S., Xanthopoulos A., Ramlawi B. (2026). Surgical judgment in the age of artificial intelligence. J. Thorac. Cardiovasc. Surg..

[7] Engelhardt S., Kostiuchik G., Bezak B., Chacko J., Daeter E., Fallouh H., Grieshaber P., Hussein N., Meyer A., Quattroni P. (2026). Current and Future View on Artificial Intelligence in Cardiothoracic Surgery. Eur. J. Cardiothorac. Surg..

[8] Benedetto U., Dimagli A., Sinha S., Cocomello L., Gibbison B., Caputo M., Gaunt T., Lyon M., Holmes C., Angelini G.D. (2022). Machine learning improves mortality risk prediction after cardiac surgery: Systematic review and meta-analysis. J. Thorac. Cardiovasc. Surg..

[9] Sinha S., Dong T., Dimagli A., Vohra H.A., Holmes C., Benedetto U., Angelini G.D. (2023). Comparison of machine learning techniques in prediction of mortality following cardiac surgery: Analysis of over 220 000 patients from a large national database. Eur. J. Cardiothorac. Surg..

[10] Nashef S.A.M., Ali J. (2023). Artificial intelligence and cardiac surgery risk assessment. Eur. J. Cardiothorac. Surg..

[11] Fuchs T.K., Jones C., Breiner M. (2026). Comparison Between Artificial Intelligence-Based Models and Traditional Risk Scores for Predicting Risks in Adult Cardiothoracic Surgery: A Systematic Review. J. Surg. Res..

[12] Chen X., Dai C., Peng M., Wang D., Sui X., Duan L., Wang X., Wang X., Weng W., Wang S. (2025). Artificial intelligence driven 3D reconstruction for enhanced lung surgery planning. Nat. Commun..

[13] Geng J., Guan T., Zeng X., Cui Z., Han H., Li Y., Chen X. (2026). Artificial Intelligence-Driven Three-Dimensional Reconstruction System Reduced Unexpected Procedural Changes in Thoracic Surgery. Ann. Surg. Oncol..

[14] Liang H., Yan Z., Zhang Y., Dai K., Li H., Shen J., Li P., Jiang J., Zhang G., Zhang X. (2026). LungSurg: A Generative AI System for Segmentation and Phase Classification in Thoracoscopic Lobectomy. MedComm.

[15] Chen X., Liu J., Liang H., Chen Z., Liu Z., Zheng Y., Lian Z., Luo L., Chen W., Wu M. (2025). Digitalization of surgical features improves surgical accuracy via surgeon guidance and robotization. npj Digit. Med..

[16] Kalisnik J.M., Bauer A., Vogt F.A., Stickl F.J., Zibert J., Fittkau M., Bertsch T., Kounev S., Fischlein T. (2022). Artificial intelligence-based early detection of acute kidney injury after cardiac surgery. Eur. J. Cardiothorac. Surg..

[17] Pavlović J. (2025). Constructivist psychology principles of human-AI collaboration. Front. Psychol..

[18] Leon M., Feng R., Quiroz Flores M., Pelletier G., Bethencourt D., Shibata M., He H., Ruaengsri C. (2026). Blinded two-phase evaluation of large language models in complex cardiac surgery: Task-specific performance and human-AI collaboration. Front. Digit. Health.

[19] Wang D.Y., Ding J., Sun A.L., Liu S.G., Jiang D., Li N., Yu J.K. (2023). Artificial intelligence suppression as a strategy to mitigate artificial intelligence automation bias. J. Am. Med. Inform. Assoc..

[20] Leivaditis V., Androutsopoulou V., Panagiotopoulos I., Maniatopoulos A.A., Liolis E., Tasios K., Nikolakopoulos K., Andrikopoulou C., Skoura T., Antzoulas A. (2026). Calculated courage: Risk, responsibility, and the ethos of cardiac surgery in the age of artificial intelligence. Arch. Med. Sci. Atheroscler. Dis..

[21] Jones C., Thornton J., Wyatt J.C. (2023). Artificial intelligence and clinical decision support: Clinicians’ perspectives on trust, trustworthiness, and liability. Med. Law Rev..

[22] Platz J.J., Bryan D.S., Ferguson M.K., Naunheim K.S. (2026). Surgeon Perception of Artificial Intelligence in Thoracic Surgery: Insights From an International Survey. Ann. Thorac. Surg..

[23] Platz J.J., Bryan D.S., Naunheim K.S., Ferguson M.K. (2025). Gender-based differences in perceptions of artificial intelligence in clinical cardiothoracic surgery. JTCVS Open.

[24] Perrenoud B., Velonaki V.S., Bodenmann P., Ramelet A.S. (2015). The effectiveness of health literacy interventions on the informed consent process of health care users: A systematic review protocol. JBI Database Syst. Rev. Implement. Rep..

[25] Puyol-Antón E., Ruijsink B., Mariscal Harana J., Piechnik S.K., Neubauer S., Petersen S.E., Razavi R., Chowienczyk P., King A.P. (2022). Fairness in Cardiac Magnetic Resonance Imaging: Assessing Sex and Racial Bias in Deep Learning-Based Segmentation. Front. Cardiovasc. Med..

[26] Straw I., Rees G., Nachev P. (2024). Sex-Based Performance Disparities in Machine Learning Algorithms for Cardiac Disease Prediction: Exploratory Study. J. Med. Internet Res..

[27] Sargiotis G.C., Sergentanis T.N., Pavi E., Athanasakis K. (2024). Predictive Performance of Artificial intelligence Models on Heart and Lung Posttransplant Health Outcomes: A Systematic Review. Exp. Clin. Transplant..

[28] Hanna M.G., Pantanowitz L., Jackson B., Palmer O., Visweswaran S., Pantanowitz J., Deebajah M., Rashidi H.H. (2025). Ethical and Bias Considerations in Artificial Intelligence/Machine Learning. Mod. Pathol..

[29] Hashimoto D.A., Marwaha J.S., Lee S.A., Schwaitzberg S., Duffourc M.N. (2026). Risk and liability in the deployment of AI systems for surgery: A SAGES white paper. Surg. Endosc..

[30] Duffourc M., Møllebæk M., Druedahl L.C., Minssen T., Gerke S. (2025). Surgeons’ Perspectives on Liability for the Use of Artificial Intelligence Technologies in the United States and European Union: Results From a Focus Group Study. Ann. Surg. Open.

[31] Khullar D., Casalino L.P., Qian Y., Lu Y., Chang E., Aneja S. (2021). Public vs physician views of liability for artificial intelligence in health care. J. Am. Med. Inform. Assoc..

[32] Amann J., Vetter D., Blomberg S.N., Christensen H.C., Coffee M., Gerke S., Gilbert T.K., Hagendorff T., Holm S., Livne M. (2022). To explain or not to explain?-Artificial intelligence explainability in clinical decision support systems. PLoS Digit. Health.

[33] Knop M., Weber S., Mueller M., Niehaves B. (2022). Human Factors and Technological Characteristics Influencing the Interaction of Medical Professionals with Artificial Intelligence-Enabled Clinical Decision Support Systems: Literature Review. JMIR Hum. Factors..

[34] Park H.J. (2024). Patient perspectives on informed consent for medical AI: A web-based experiment. Digit. Health.

[35] Rose S.L., Shapiro D. (2024). An Ethically Supported Framework for Determining Patient Notification and Informed Consent Practices When Using Artificial Intelligence in Health Care. Chest.

[36] Ferrari-Light D., Merritt R.E., D’Souza D., Ferguson M.K., Harrison S., Madariaga M.L., Lee B.E., Moffatt-Bruce S.D., Kneuertz P.J. (2025). Evaluating ChatGPT as a patient resource for frequently asked questions about lung cancer surgery-a pilot study. J. Thorac. Cardiovasc. Surg..

[37] Leivaditis V., Maniatopoulos A.A., Lausberg H., Mulita F., Papatriantafyllou A., Liolis E., Beltsios E., Adamou A., Kontodimopoulos N., Dahm M. (2025). Artificial Intelligence in Thoracic Surgery: A Review Bridging Innovation and Clinical Practice for the Next Generation of Surgical Care. J. Clin. Med..

[38] Leivaditis V., Beltsios E., Papatriantafyllou A., Grapatsas K., Mulita F., Kontodimopoulos N., Baikoussis N.G., Tchabashvili L., Tasios K., Maroulis I. (2025). Artificial Intelligence in Cardiac Surgery: Transforming Outcomes and Shaping the Future. Clin. Pract..

[39] Cardaioli F., Fovino L.N., Tarantini G. (2023). Prime-Time for Heart(ificial)-Team?. Am. J. Cardiol..

[40] European Parliament & Council of the European Union (2024). Regulation (EU) 2024/1689 Laying Down Harmonised Rules on Artificial Intelligence (Artificial Intelligence Act).

[41] U.S. Food and Drug Administration (2026). Clinical Decision Support Software: Guidance for Industry and Food and Drug Administration Staff. https://www.fda.gov/regulatory-information/search-fda-guidance-documents/clinical-decision-support-software.

[42] U.S. Food and Drug Administration (2025). Marketing Submission Recommendations for a Predetermined Change Control Plan for Artificial Intelligence-Enabled Device Software Functions: Guidance for Industry and Food and Drug Administration Staff. https://www.fda.gov/regulatory-information/search-fda-guidance-documents/marketing-submission-recommendations-predetermined-change-control-plan-artificial-intelligence.

[43] Adegunle F., Chhatwal K., Arab S., Alabdaljabar M.S., Raslan M.A., Sayed O., Goldsweig A.M. (2026). Bias and Oversight in Clinical AI: A Review of Decision Support Tools and Equity Frameworks. J. Gen. Intern. Med..

[44] Shaw D., Lorenzini G., Arbelaez Ossa L., Eckstein J., Steiner L., Elger B.S. (2025). When and what patients need to know about AI in clinical care. Swiss Med. Wkly..

